# Migrating Foreign Body in the Heart

**DOI:** 10.7759/cureus.25294

**Published:** 2022-05-24

**Authors:** Anthony Lemaire, Raymond Kennedy, Hirohisa Ikegami, Manabu Takebe, Gengo Sunagawa, Mark J Russo, Leonard Lee

**Affiliations:** 1 Cardiac Surgery, Rutgers Robert Wood Johnson Medical School, New Brunswick, USA; 2 Cardiothoracic Surgery, Robert Wood Johnson University Hospital, New Brunswick , USA

**Keywords:** device migration, catheter migration, right ventricle (rv), heart surgery, foreign bodies

## Abstract

Foreign bodies in the heart are a rare condition and an exact mechanism for this occurrence has not been well described. These objects can reach the heart by direct penetration due to local trauma or through intravenous migration or may remain in the heart after medical procedures. The most common foreign bodies that reach the heart are bullets and shrapnel. The purpose of this study is to review a case where a patient injected himself with recreational drugs. The needle subsequently dislodged from the syringe and migrated into the heart.

## Introduction

Migrating foreign bodies into the heart are a rare condition that has not been well reported. These objects can reach the heart by direct penetration due to trauma or through intravenous migration or after medical procedures. The most common foreign bodies that reach the heart are bullets and shrapnel. There are parts of catheters and needles that can reach the right heart by migration through the venous system. The main radiographic modalities for detecting foreign bodies in the heart are x-ray-based methods [1]. Additional imaging studies such as computerized tomography (CT) scans and echocardiography can provide more accurate details. The purpose of this study is to review a case where a patient injected himself with recreational drugs. The needle subsequently dislodged from the syringe and migrated into the heart.

## Case presentation

**Figure 1:**
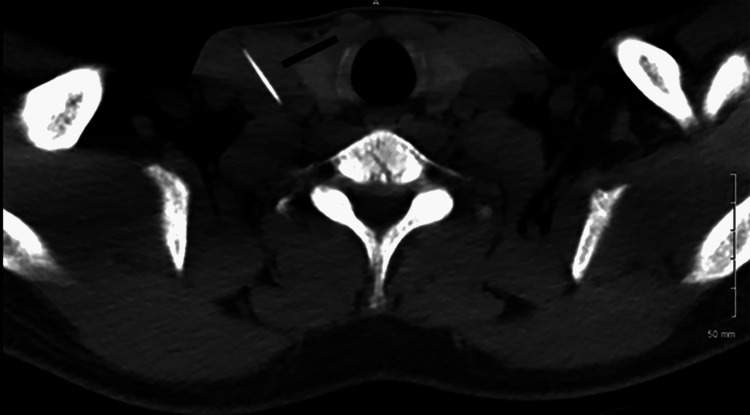
Needle within the right internal jugular vein in the neck.

This is the case of a 32-year-old man with a history of intravenous drug abuse with recent use at 3 am on the day of admission. He reported using a 3-cm-long, 25-gauge needle to inject heroin into his right internal jugular vein. Upon injecting the syringe, the patient noticed that the needle was not present and retained in his neck. He presented to the emergency room and a CT scan of the chest showed a radiopaque object at the injection site, tracking medial to the right internal jugular vein (Figure 1). The patient was taken to the operating room by the vascular surgery team for attempted removal of the needle. Unfortunately, despite an aggressive exploration no needle was found. Additional imaging was ordered and a CT scan of the chest showed a foreign object in the heart.

**Figure 2:**
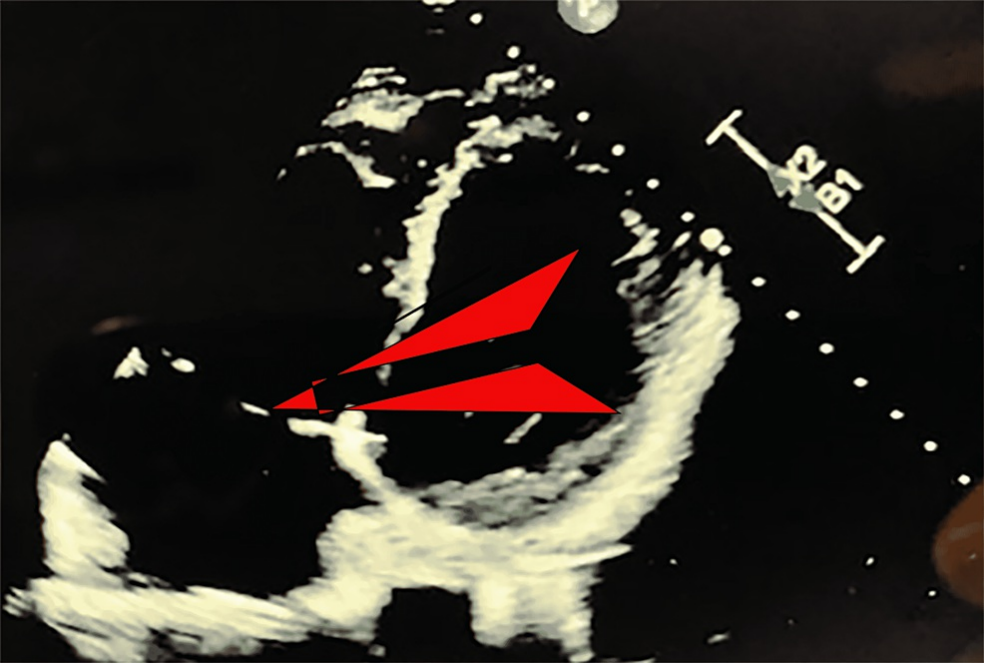
Dislodged needle below the tricuspid valve and imbedded in the interventricular septum.

**Figure 3:**
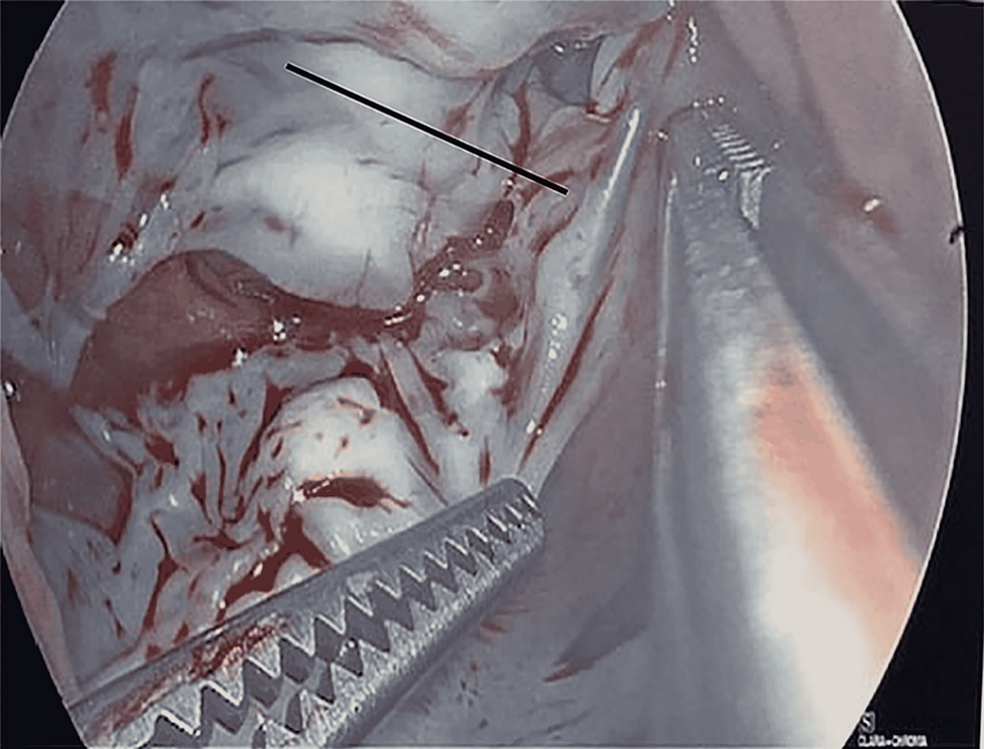
Dislodged needle below the septal leaflet of the tricuspid valve.

**Figure 4:**
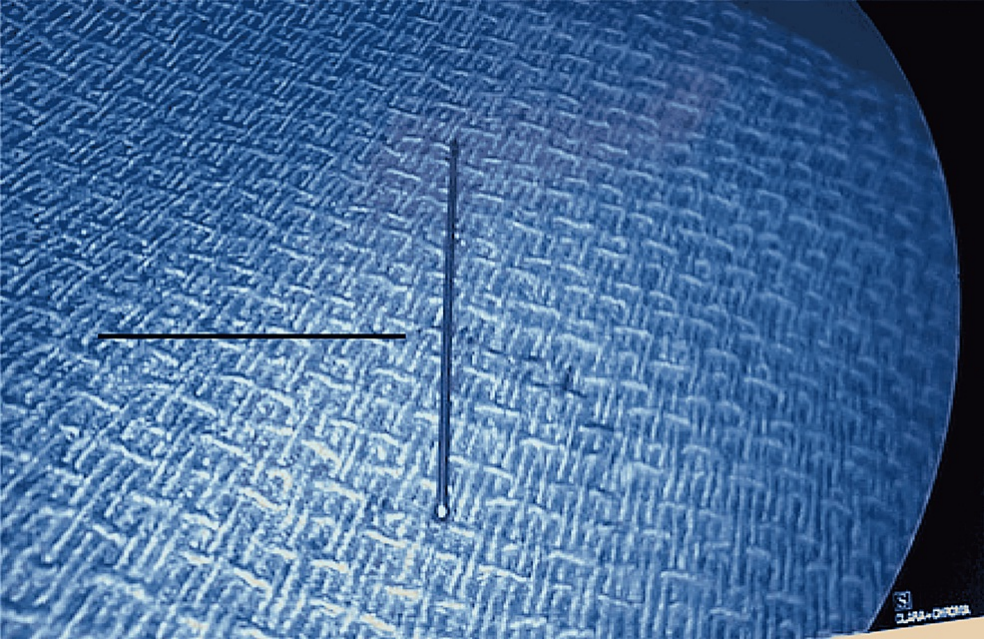
Needle removed from the heart.

At this time, cardiothoracic surgery was consulted. After review of the CT scan of the chest and intraoperative transesophageal echocardiogram (Figure 2), which confirmed the needle in the heart, an exploration and removal of the needle was initiated. The needle was specifically identified in the right atrium within the septal leaflet of the tricuspid valve. There was no injury to the leaflet and no resulting valvular pathology. After a median sternotomy, the patient was placed on cardiopulmonary bypass with bicaval cannulation. The right atrium was opened, and the needle was clearly seen below the septal leaflet of the tricuspid valve (Figure 3). The needle was carefully removed with care to prevent injury to the tricuspid valve (Figure 4). The right atrium was then closed and the patient was taken off the cardiopulmonary bypass machine. He subsequently did well and was discharged home on postoperative day number 4. He returned to clinic and was doing well. 

## Discussion

This case report reviews a patient who had a 3-cm needle dislodged into his venous system and implanted into his right atrium. A migrating foreign body in the heart is a unique presentation to the emergency room. Although there have been reports of patients having objects in their hearts, this case has several unique aspects. First, this patient injected himself with the needle that migrated into his right atrium, in contrast to most presentations where patients are victims of trauma or iatrogenic events. Second, the patient was taken to the operating room twice with two different surgical services, vascular and cardiothoracic surgery, for attempted needle removal. Third, a needle being dislodged is not very common in contrast to the more common objects although it has been reported [2,3]. For example, inferior vena cava (IVC) filters or vascular stents are more commonly migrated in comparison to needles [4].

Traditionally, patients who have migration of their IVC ﬁlters are usually symptomatic (12 of 14 cases), and may occur a long time after the insertion of the ﬁlter - in one patient four years following the procedure [4]. It has contributed to the cause of cardiorespiratory arrest in three out of 14 patients [5] and was associated with arrhythmias and death in two out of 14 patients [6], and led to the majority of death (50%) in the reported 104 patients. Moreover, larger devices than catheters and migrated stents can cause symptoms during the first year after placement. The larger foreign object can cause cardiac injury including aorto-right atrial ﬁstulas, pericardial effusions, and valvular injuries [7,8]. These findings of migrating objects contributing to injury of the heart should be a lesson to not only physicians who perform procedures but also medical doctors who do not perform procedures. 

## Conclusions

The case report highlights the dangers of dislodged and migrating objects in the body. A 3-cm needle ultimately led to two surgical procedures for extraction. In addition, the diagnostic studies identifying the foreign body were critical in assisting with surgical removal. A blind surgical exploration without the use of focused imaging would be very difficult. Finally, migrating foreign bodies although rare can lead to serious consequences. The prompt removal of these objects is warranted for the safety of the patients.
